# Moderating effects of socioeconomic status and geographical location on the Health4Life school-based intervention

**DOI:** 10.1016/j.pmedr.2024.102855

**Published:** 2024-08-13

**Authors:** Lyra Egan, Lauren A. Gardner, Nicola C. Newton, Siobhan O’Dean, Katrina E. Champion

**Affiliations:** The Matilda Centre for Research in Mental Health and Substance Use, The University of Sydney, Australia

**Keywords:** Sociodemographic disadvantage, Adolescent health, Lifestyle behaviours, Prevention, eHealth, School-based intervention, Latent growth modelling

## Abstract

**Objective:**

This study evaluated the moderating effects of socioeconomic status (SES) and geographical location on the efficacy of an eHealth school-based multiple health behaviour change intervention – *Health4Life* – in targeting alcohol and tobacco use, dietary intake, knowledge, behavioural intentions, and psychological distress over 24-months.

**Methods:**

Data from the *Health4Life* cluster-randomised controlled trial conducted from 2019 to 2021 in 71 Australian secondary schools were analysed (N=6639; baseline age 11-14yrs). Schools were from metropolitan (89%) and regional (11%) areas, and participants’ SES was classified as low (15%), mid (37%), and high (48%) relative to the study population. Primary outcomes included alcohol and tobacco use, and a composite indicator of poor diet. Secondary outcomes were knowledge, behavioural intentions, and psychological distress. Latent growth models assessed moderating effects of SES and geographical location on between-group change over 24-months.

**Results:**

Geographical location moderated the intervention’s effect on odds of reporting a poor diet (OR = 1.79, 95% CI = 1.32–2.43, p < 0.001) and diet-related behavioural intentions (OR = 0.71, 95% CI = 0.56–0.89, p = 0.024) over time. Subset analyses indicated that intervention participants in regional areas had higher odds of reporting a poor diet (OR = 1.61, 95% CI = 1.13–2.29, p = 0.008), while those in metropolitan areas had higher odds of improving diet-related behavioural intentions (OR = 1.13, 95% CI = 1.01–1.27, p = 0.041), compared to the control group. No other significant moderation effects were observed.

**Conclusions:**

While significant disparities were generally not observed, the geographical differences in intervention effects on diet and diet-related intentions suggest that co-designed and tailored approaches may benefit disadvantaged adolescents to address the disproportionately high rates of lifestyle risk behaviours among these priority populations.

## Highlights

• Universal eHealth school-based multiple behaviour change RCT among adolescents.
• Latent growth models assessing SES and geographical location as moderators of RCT.
• Diet and diet-related intentions varied by geographical location over 24-months.
• Sociodemographic factors can influence intervention efficacy.
• Tailored approaches may be beneficial for tackling adolescent risk behaviour disparities.

## 1 Introduction

Sociodemographic inequalities, including socioeconomic status (SES) and geographical location (e.g., urban versus rural areas), significantly contribute to chronic disease burden and are critical determinants of access to resources and health outcomes in society (Australian Institute of Health and Welfare, 2023, Robards et al., 2018). Adolescents from low SES and geographically remote contexts are particularly susceptible to this burden (Australian Institute of Health and Welfare, 2021). The 2018 Australian Burden of Disease Study (Australian Institute of Health and Welfare, 2021), reports a progressive increase in disability-adjusted life years from affluent to disadvantaged socioeconomic areas, and from major cities to remote areas, with 1.6 and 1.4 times higher rates, respectively. Despite unique differences between adolescents of low SES and geographically remote backgrounds, both groups face similar challenges in achieving health equity. Therefore, the term “disadvantaged” is used to describe these individuals in this context. Disadvantaged adolescent populations encounter various obstacles that impede their access to health, including stigmatisation, lack of social support, limited access to affordable services, and education opportunities (Australian Institute of Health and Welfare, 2023, Robards et al., 2018).

Disadvantaged adolescent populations, both in Australia and globally, exhibit higher rates of modifiable lifestyle risk behaviours such as poor diet, alcohol use, and tobacco smoking (Australian Institute of Health and Welfare., 2020, Warren et al., 2017, Wiggins et al., 2020). These may continue into adulthood, increasing the risk of chronic disease and associated burden, especially when they occur together (Krokstad et al., 2017). Adolescence is a critical developmental period (Sawyer et al., 2012), characterised by increased risk-taking tendencies, including experimentation with alcohol and smoking (Degenhardt et al., 2016), and consuming unhealthy foods (Australian Institute of Health and Welfare, 2018). Clustering or the co-occurrence of these behaviours in adolescence is common (Uddin et al., 2020), and linked with adverse outcomes, including obesity (Bardach et al., 2023), reduced quality of life (Hoare et al., 2019) and mental ill-health (Champion et al., 2018, Gardner et al., 2022a). Indeed, research has reported that adolescents with a history of early-life low SES often face co-occurring adverse health and educational challenges (Villadsen et al., 2023). Therefore, prioritising prevention during adolescence is crucial for promoting healthy behaviours, mitigating the risk of chronic diseases in adulthood, and addressing health disparities experienced by this vulnerable population.

Electronic health (eHealth) interventions hold promise in benefiting disadvantaged adolescents by providing accessible and low-cost resources that can increase student engagement and implementation fidelity (Champion et al., 2019). Multiple systematic reviews have demonstrated the efficacy of universal eHealth interventions in preventing and addressing poor diet (Kemp et al., 2021), tobacco smoking (Taylor et al., 2017), and/or reducing alcohol use (Kazemi et al., 2021) among adolescents. Although, a recent meta-analysis of universal school-based prevention eHealth interventions targeting multiple health risk behaviours found them ineffective in preventing alcohol consumption or smoking and reducing fat, sugar-sweetened beverages (SSB), or snack consumption (Champion et al., 2019). The review recommended focusing on skill development and social influence and competence theories for improved outcomes. Despite these mixed findings, evidence supports the effectiveness of universal eHealth interventions in preventing or delaying the onset of modifiable health risk behaviours (Newton et al., 2017, Qiu et al., 2022). However, their effectiveness for disadvantaged adolescents is not as well-known, with only one systematic review published on eHealth prevention interventions targeting poor diet, alcohol use, and tobacco smoking among disadvantaged adolescents (Egan et al., 2023). The review indicated that eHealth interventions can be effective in targeting poor diet (e.g., decreasing SSB consumption) and alcohol use (e.g., reducing binge drinking at 1-month follow-up among intervention completers) among disadvantaged adolescents. However, it also acknowledged limitations due to the scarcity of published studies on this topic, with only 15 publications assessing 14 interventions eligible for inclusion.

The Australian *Health4Life* initiative is an innovative eHealth school-based intervention targeting multiple lifestyle risk behaviours among adolescents: alcohol use, tobacco smoking, poor diet, physical inactivity, poor sleep, and sedentary recreational screen time (Teesson et al., 2020). Co-designed with young people, *Health4Life* incorporates personalised feedback and is grounded in social influence, social cognitive, and self-determination theories (Champion et al., 2020). Results from a cluster randomised controlled trial (RCT) of *Health4Life* found significant effects on reducing behavioural intentions to try alcohol and tobacco at post-intervention (O’Dean et al., Under review), improving mental health outcomes (Smout et al., 2024) and knowledge about chronic disease risk factors over 24-months (Champion et al., 2023). However, the RCT reported no significant intervention effects on modifying alcohol or tobacco use, poor diet, physical inactivity, poor sleep or screen time across the entire sample (Champion et al., 2023). Despite *Health4Life*’s positive reception by students (74.8%) and teachers (84%), and the significant improvement in knowledge, this did not translate into behaviour change. *Health4Life* baseline data (n = 6639 11–14 year-olds across NSW, WA, and QLD) revealed socio-demographic disparities in diet, alcohol, and tobacco use (Champion et al., 2021). For instance, students from regional areas were more likely to use alcohol than those from major cities, and students with lower SES were more likely to use alcohol and tobacco and have poorer diets than their peers with middle to upper SES. Given these disparities we expect that *Health4Life*’s efficacy may differ in these subgroups, potentially due to distinct challenges influencing their health behaviours differently. Tailored approaches may be needed, however, evidence is currently unclear.

Nonetheless, considering the significant influence of SES and geographical factors on health-related behaviours, it is essential to examine their impact on the efficacy of interventions such as *Health4Life*. This study aims to evaluate the moderating effects of SES and geographical location on the efficacy of the *Health4Life* intervention in reducing alcohol and tobacco use, improving dietary intake, knowledge of chronic disease risk behaviours, behavioural intentions, and reducing psychological distress.

## 2 Methods

### 2.1 Participants and procedure

The current study uses baseline to 24-month follow-up data from the *Health4Life* study (Teesson et al., 2020), a cluster RCT in 71 secondary schools across Australia. Recruitment details have been reported previously (Champion et al., 2023). Briefly, 71 schools were block randomised (1:1) by a biostatistician independent to recruitment to either the *Health4Life* intervention (N=36) or active control group (usual health education; N=35). Randomisation was stratified by school location (state/region) and gender composition (coeducational, mostly female [>60 %], or mostly male [>60 %]). It was not possible to blind students, teachers, and researchers to group assignment, as is standard with school-based interventions.

The study’s parental consent approaches varied based on the ethical requirements of the schools involved. While 40 schools provided an opt-out option, 31 required active written and oral consent (i.e., opt-in). All students provided active written consent to participate in the study.

The intervention group received *Health4Life*, consisting of six web-based modules delivered during health education lessons, ideally once per week. Based on social influence, social cognitive, and self-determination theories to prevent multiple lifestyle risk behaviours, key behaviour change techniques are integrated into 20-minute interactive cartoon storylines with characters similar in age to grade 7 students (Champion et al., 2020). These cartoons, the core component of *Health4Life*, impart evidence-based information about health and social consequences of poor diet, alcohol use, tobacco smoking, physical inactivity, poor sleep, and sedentary recreational screen time, while also promoting resistance skills, normative education, and autonomous motivation. Students complete short online quizzes after each module, and factsheets for teachers and students are available to reinforce the content. *Health4Life* is supplemented by web-based tailored feedback on adherence to national health guidelines, optional online or teacher-led activities, and a smartphone app designed to encourage self-monitoring of behaviours and goal setting. Control schools delivered usual health education, approximately once a week.

Participants completed self-report online surveys during class at four time points: baseline (2019); immediately following the intervention (2019); 12-months after baseline (2020); 24-months after baseline (2021). To maximise retention, two participants from each school were randomly allocated a AUD$100 gift voucher for completing the surveys.

This trial was prospectively registered with the Australian New Zealand Clinical Trials Registry (ACTRN12619000431123) and adheres to CONSORT guidelines.

### 2.2 Measures

#### 2.2.1 Sociodemographic factors

Students provided self-reported information on gender, age, SES and geographical location. SES was categorised into lower, middle and upper groups based on Family Affluence III ridit scores (Elgar et al., 2017), and geographical location was classified as metropolitan or regional based on the Australian Statistical Geography Standard Remoteness Structure (Australian Bureau of Statistics, 2021).

#### 2.2.2 Primary outcomes

Diet: The Student Physical Activity and Nutrition Survey (SPANS) measured the consumption of SSBs, fruit, vegetables, and discretionary foods (Hardy et al., 2016). A composite indicator of poor diet was then calculated. Individuals were classified as having a poor diet if they reported high SSB consumption (5–6 cups/week or 1 or more cups/day of SSB) or met two or more of the following conditions: consuming fewer than two servings of fruit per day, consuming fewer than five servings of vegetables per day, or consuming more than one serving of discretionary food items per day. The cut-offs for fruit and vegetable intake were based on the Australian Dietary Guidelines (National Health and Medical Research Council, 2013), while nutritionists and health recommendations guided SSB and discretionary food variables.

Alcohol: A single item measured participants’ consumption of a full standard drink by asking participants, “Have you had a full standard alcoholic drink in the past 6 months?” (0 = No, 1 = Yes). To facilitate participants’ responses, they were shown a pictorial chart displaying the standard drink quantities of various types of alcoholic beverages and sizes.

Tobacco: A single item measure from the Youth Risk Behaviour Survey assessed tobacco use with participants asked, “In the past 6 months, have you tried cigarette smoking, even one or two puffs?” (0 = No, 1 = Yes) (Brener et al., 2013).

#### 2.2.3 Secondary outcomes

Knowledge: A 20-item scale assessed participants’ knowledge of *Health4Life* study content, including awareness of chronic disease risk factors, alcohol guidelines, prevalence of alcohol and tobacco use among young Australians, and the impact of six lifestyle risk behaviours on physical and mental health. Items were presented as “True”, “False”, “Don’t Know” statements, and scores were totalled to produce an overall knowledge score.

Behavioural Intentions: Participants self-reported their intentions to participate in or modify behaviours relating to poor diet, alcohol and tobacco use. Alcohol intentions were evaluated using established measures (Newton et al., 2012), with items for tobacco and poor diet (specifically SSBs including energy drinks, soft drinks, sports drinks, or cordial) adapted from these measures. Participants rated their likelihood of trying alcohol and tobacco on a scale from 0 (very unlikely) to 4 (very likely). For SSBs, they indicated their intention to replace them with water on a scale from 0 (not at all true of me) to 3 (very true of me) on all or most days over the next three months. Responses were then converted into binary variables for subsequent analyses in this study. Scores of 0–2 for alcohol and tobacco questions indicated no intention to engage in alcohol use or tobacco smoking, while scores of 3–4 indicated an intention to do so. Regarding SSBs, responses of 0–1 were recoded as no intention to replace SSB consumption with water, while responses of 2–3 indicated an intention to make the substitution.

Psychological Distress: The Kessler 6 (K6) scale, a widely used and validated measure, assessed psychological distress among adolescents by evaluating six symptoms experienced by individuals over the past four weeks (Furukawa et al., 2003, Mewton et al., 2016). These symptoms include feeling nervous, hopeless, or restless. Participants rated each symptom on a 5-point Likert scale ranging from “none of the time” to “all of the time”. Scores were totalled to create a composite score, with higher scores indicating greater psychological distress.

### 2.3 Statistical analysis

A post-hoc exploratory moderation analysis was chosen for the current study. Latent growth curve models (LGMs) in Mplus (v 8.4. (Muthén & Muthén, 2017)) were used to evaluate the moderating effects of SES and geographical location on primary and secondary outcomes over 24-month post baseline assessments. Various LGMs were used based on the distribution of the outcome variables, including binary, continuous, or ordinal models. To determine the optimal time structure and interpretation of slope estimates for each outcome, we explored various specifications of time scores, including linear, quadratic, and freely estimated, on unconditional LGMs. We compared model fit using Akaike information criterion (AIC), Bayesian information criterion (BIC), and sample-size adjusted BIC. Further details regarding model interpretation is provided in the appendices on page 8. The analysis applied the full-information maximum likelihood (FIML) estimation within the LGMs, aligning with intention-to-treat principles to handle missing data. FIML uses all available information when estimating parameters, and it is recognised for its superiority over conventional methods within the context of LGMs (Schafer & Graham, 2002). To account for the effect of testing multiple outcomes, the Benjamini-Hochberg approach, also known as the false discovery rate control, was applied (Benjamini & Hochberg, 1995). Unlike more conservative methods such as the Bonferroni correction, the Benjamini-Hochberg approach offers increased power to identify true associations when testing multiple outcomes. To interpret statistically significant interactions (p < 0.05), we conducted subset analyses that investigated the main effects of intervention within each level of the moderating variables (i.e., SES or geographical location).

### 2.4 Ethics

The *Health4Life* study was approved by the University of Sydney (2018/882), the University of Queensland (2019000037), Curtin University (HRE2019–0083), and ethics committees in the relevant school sectors.

## 3 Results

### 3.1 Descriptive statistics

A total of 6,639 students from 71 schools participated in the baseline survey, constituting the analysed sample for this study. The students had a mean age of 12.7 years (SD 0.50; range 11–14 years). In terms of gender, 49.9% (n = 3,311) identified as male, 48.3% (n = 3,204) identified as female, 0.5% (n = 30) identified as non-binary or gender fluid, 0.1% (n = 9) had a different gender identity, and 1.0% (n = 69) preferred not to disclose their gender identity. The sample included students from diverse socioeconomic backgrounds, with 15% classified as low SES, 37% as mid SES, and 48% as high SES. Furthermore, the sample represented both metropolitan and regional areas, with 89% residing in metropolitan areas and 11% in regional areas (Table 1). Retention rates and the number of students who completed follow-up surveys are reported in Appendix Fig. 1.

**Table 1:** Baseline (2019) sample characteristics of adolescents participating in the Australian Health4Life Study.

Characteristic											
	Socioeconomic status n (%)						Geographical location n (%)				
	Low SES: Control	Low SES: Health4Life	Mid SES: Control	Mid SES: Health4Life	High SES: Control	High SES: Health4Life	Metropolitan: Control	Metropolitan: Health4Life	Regional: Control	Regional: Health4Life	
	351 (5.3)	558 (8.4)	1005 (15.1)	1204 (18.1)	1336 (20.1)	1560 (23.5)	2650 (39.9)	3303 (49.8)	380 (5.7)	306 (4.6)	**Total**
**Age (mean, SD)**	12.66 (0.49)	12.65 (0.51)	12.63 (0.51)	12.67 (0.49)	12.64 (0.51)	12.66 (0.49)	12.64 (0.51)	12.65 (0.50)	12.64 (0.49)	12.79 (0.49)	12.7 years (0.50)
**Gender n (%)**											
Male	178 (50.9)	330 (59.1)	470 (47.0)	659 (54.7)	612 (45.8)	702 (45.0)	1239 (46.9)	1671 (50.7)	207 (54.6)	194 (63.6)	3,311 (49.9)
Female	164 (46.9)	217 (38.9)	510 (50.9)	520 (43.2)	705 (52.8)	844 (54.1)	1354 (51.2)	1574 (47.8)	167 (44.1)	108 (35.4)	3,204 (48.3)
Non-binary or gender fluid	3 (0.9)	5 (0.9)	8 (0.8)	4 (0.3)	3 (0.2)	4 (0.3)	15 (0.6)	14 (0.4)	1 (0.3)	0 (0.0)	30 (0.5)
Different gender identity	0 (0.0)	1 (0.2)	1 (0.1)	3 (0.2)	2 (0.1)	1 (0.1)	4 (0.2)	4 (0.1)	0 (0.0)	1 (0.3)	9 (0.1)
Preferred not to disclose	5 (1.4)	5 (0.9)	12 (1.2)	18 (1.5)	14 (1.0)	9 (0.6)	30 (1.1)	33 (1.0)	4 (1.1)	2 (0.7)	69 (1.0)

**State n (% of participants)**											
New South Wales	220 (62.7)	355 (63.6)	487 (48.5)	645 (53.6)	639 (47.8)	929 (59.6)	1392 (52.5)	1808 (54.7)	103 (27.1)	233 (76.1)	37 schools (53.2)
Queensland	56 (16.0)	141 (25.3)	214 (21.3)	412 (34.2)	332 (24.9)	493 (31.6)	655 (24.7)	1133 (34.3)	0 (0.0)	0 (0.0)	18 schools (26.9)
Western Australia	75 (21.4)	62 (11.1)	304 (30.2)	147 (12.2)	365 (27.3)	138 (8.8)	603 (22.8)	362 (11.0)	277 (72.9)	73 (23.9)	16 schools (19.8)

### 3.2 Moderation effects in latent growth curve models

#### 3.2.1 Conditional growth curve models

**Fig. 1:**
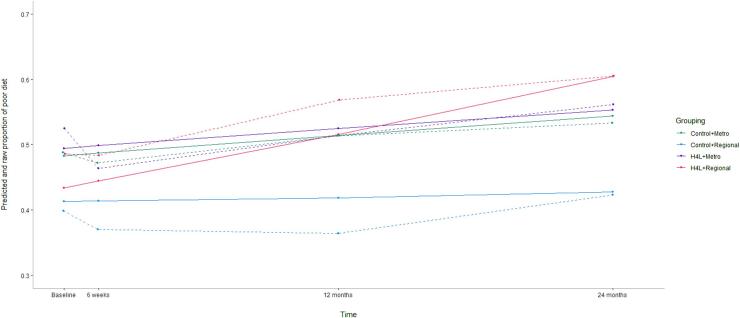
Predicted and raw proportion of poor diet among metropolitan and regional participants in the Australian Health4Life Study. Solid lines are raw values and dotted lines are predicted values.

**Fig. 2:**
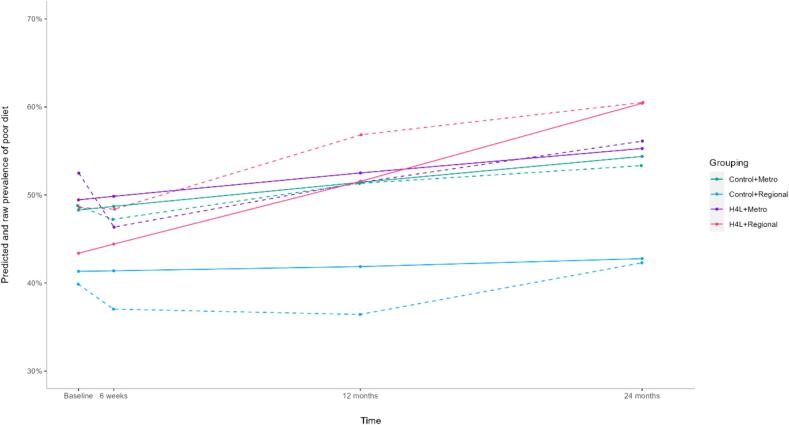
Predicted and raw proportion of intentions to swap SSB for water among metropolitan and regional participants in the Australian Health4Life Study. Solid lines are raw values and dotted lines are predicted values.

There was a significant moderating effect of geographical location on intervention effectiveness for poor diet (OR = 1.79, 95% CI = 1.32–2.43, p < 0.001) (Fig. 1). Subset analyses revealed for those who resided regionally, there was greater growth in the odds of having a poor diet in the intervention group compared to the control (OR = 1.61, 95% CI = 1.13–2.29, p = 0.008), and little evidence of a difference between groups for those residing in metropolitan cities (OR = 0.99, 95% CI = 0.86–1.14, p = 0.915) (Appendix Table 4). There was little evidence supporting a moderation effect of SES on intervention effectiveness for poor diet (OR = 0.99, 95% CI = 0.87–1.13, p = 0.856) (Table 3). In addition, geographical location significantly moderated the intervention effectiveness in promoting behavioural intentions to swap SSB for water (OR = 0.71, 95% CI = 0.56–0.89, p = 0.024 (Fig. 2)). Subset analyses revealed for those who resided in metropolitan cities, there was a significantly greater increase in the odds of intending to swap SSB for water in the intervention group compared to the control (OR = 1.13, 95% CI = 1.01–1.27, p = 0.041), and little evidence of a difference between groups for those who resided regionally (OR = 0.97, 95% CI = 0.72–1.32, p = 0.857). There was little evidence of a moderation effect of SES on the intervention effectiveness in targeting diet-related behavioural intentions at 24-months (OR = 1.03, 95% CI = 0.92–1.15, p = 0.685).

There was little evidence for a moderation effect of SES or geographical location on the average change in odds of alcohol (OR = 0.81, 95% CI = 0.52–1.25, p = 0.489) or tobacco use (OR = 0.60, 95% CI = 0.37–0.95, p = 0.124) in the past 6-months, and alcohol-related (OR = 0.98, 95% CI = 0.81–1.19, p = 0.856) and tobacco-related behavioural intentions (OR = 0.86, 95% CI = 0.68–1.08, p = 0.457) over the 24-months. Likewise, over the 24-month duration there were no moderation effects of SES on the mean knowledge scores of chronic disease risk factors (0.08, SE=0.09, p = 0.502) or psychological distress (−0.13, SE=0.13, p = 0.489), nor of geographical location on the mean knowledge scores of chronic disease risk factors (−0.42, SE=0.26, p – 0.365) or psychological distress (0.33, SE=0.36, p = 0.495) (Table 2, Table 3).

**Table 2:** Summary of logistic latent growth parameters, CIs and SE investigating the effects of SES on moderating the odds of study outcomes from the Australian Health4Life Study.

	Intercept		Slope	
Logistic latent growth model				
	OR (CI)	p	OR (CI)	p
Poor diet	1.13 (0.88, 1.47)	0.336	0.99 (0.87, 1.13)	0.856
Alcohol	0.86 (0.52, 1.42)	0.554	0.81 (0.52, 1.25)	0.489
Tobacco	0.78 (0.43, 1.44)	0.440	0.60 (0.37, 0.95)	0.124
Diet intentions	0.97 (0.81, 1.16)	0.743	1.03 (0.92, 1.15)	0.685
Alcohol intentions	0.62 (0.39, 0.99)	0.046	0.98 (0.81, 1.19)	0.856
Tobacco intentions	0.88 (0.57, 1.38)	0.587	0.86 (0.68, 1.08)	0.457

Continuous latent growth model				
	b(SE)	p	b(SE)	p
Knowledge	−0.43(0.19)	0.022	0.08(0.09)	0.502
Psychological Distress	−0.11(0.25)	0.671	−0.13(0.13)	0.489

**Table 3:** Summary of logistic latent growth parameters, CIs and SE investigating the effects of Metropolitan-Regional on moderating the odds of study outcomes from the Australian Health4Life Study.

	Intercept		Slope	
Logistic latent growth model				
	OR (CI)	p	OR (CI)	p
Poor diet	1.13 (0.87, 1.46)	0.369	1.79 (1.32, 2.43)	<0.001
Alcohol	0.84 (0.51, 1.39)	0.501	2.53 (0.44, 14.48)	0.489
Tobacco	0.80 (0.43, 1.48)	0.473	5.92 (1.45, 24.18)	0.069
Diet intentions	0.96 (1.15, 1.27)	0.666	0.71 (0.56, 0.89)	0.024
Alcohol intentions	0.60 (0.38, 0.97)	0.037	0.76 (0.44, 1.33)	0.489
Tobacco intentions	0.83 (0.55, 1.27)	0.392	2.01 (0.75, 5.38)	0.437

Continuous latent growth model				
	b(SE)	p	b(SE)	p
Knowledge	−0.38(0.19)	0.046	−0.42(0.26)	0.365
Psychological Distress	−0.10(0.25)	0.679	0.33(0.36)	0.495

## 4 Discussion

This is the first study to examine the moderating effects of SES and geographical location on the effectiveness of the *Health4Life* intervention in reducing alcohol and tobacco use, improving dietary intake, knowledge of chronic disease risk behaviours and behavioural intentions, and reducing psychological distress over 24-months. Overall, the study found little evidence for a moderation effect, with the exception of diet-related outcomes by geographical location. This is consistent with the primary outcomes of the *Health4Life* RCT, which revealed no significant intervention effects on alcohol use, tobacco smoking and poor diet across the entire sample (Champion et al., 2023).

Importantly, *Health4Life* draws from social influence, social cognitive, and self-determination theories (Champion et al., 2020), which aligns with recommendations for eHealth interventions among adolescents of low SES or of geographically remote backgrounds (Egan et al., 2023). However, *Health4Life* was not specifically designed to address the unique experiences of disadvantaged adolescents, who often encounter structural barriers including limited access to resources, support, and economic constraints (Australian Institute of Health and Welfare, 2023, Robards et al., 2018). These barriers create a more challenging environment for adopting and maintaining positive health behaviours compared to their more advantaged peers. For instance, healthy food options are less affordable and less available in low SES and regional areas (Love et al., 2018), and there may be lower levels of social support (Gautam et al., 2023), both of which are crucial for sustaining healthy behaviours. Moreover, *Health4Life*’s foundational principles assume a level of autonomy and resource availability that disadvantaged adolescents may not have e.g. financial constraints limiting control over health choices. While the theoretical underpinnings of *Health4Life* are sound, their practical application may be undermined if positive health behaviours are not modelled or encouraged (Bandura, 2004) which may be the reality in disadvantaged adolescents’ environments. Together this may explain why *Health4Life* was limited in effectively modifying alcohol and tobacco use, knowledge, alcohol- and tobacco-related behavioural intentions, and psychological distress in this population.

The present findings suggest that, generally, outcomes were not influenced by SES and geographical location. Instead, the intervention’s effects may be attributed to other underlying mechanisms. For instance, despite the myriad of challenges linked to varying SES and regional contexts, *Health4Life* maintained a positive impact on adolescents’ knowledge of chronic disease risk behaviours, potentially due to other factors such as personal motivation or learning styles. The absence of significant moderation effects on other outcomes, including alcohol and tobacco use, suggests that *Health4Life*’s impact on these behaviours may vary depending on individual characteristics and contextual factors, such as family dynamics and social support that could be targeted through tailored interventions. For example, interventions may target the home environment by involving parents. Additionally, including peers in the intervention process may help create a supportive network. These refinements may benefit *Health4Life*’s efficacy for all participants, and to further enhance *Health4Life*’s efficacy for low SES and regional populations, addressing structural barriers and other social determinants that could influence health behaviours is essential. Given disadvantaged adolescents exhibit higher rates of these lifestyle risk behaviours than their more advantaged peers (Australian Institute of Health and Welfare., 2020, Warren et al., 2017, Wiggins et al., 2020), this tailoring may lead to more significant and equitable health improvements among these populations. This highlights the importance of co-design and tailoring interventions to disadvantaged populations (Egan et al., 2023). Emerging interventions such as Just-in-Time-Adaptation, which use real-time data to provide personalised support through digital platforms, may also be beneficial for this population as they are easily accessible, individually tailored and practical (Partridge & Redfern, 2018).

Diet-related outcomes are an exception to the null findings. *Health4Life* improved diet-related behavioural intentions for those residing in metropolitan areas, but not regional areas. However, this did not translate into dietary behaviour change, contrasting with a separate study that improved both behavioural intentions and fruit and vegetable consumption (Kothe et al., 2012). This discrepancy challenges the Theory of Planned Behaviour, which posits that intention is a proximal determinant of behaviour (Bosnjak et al., 2020). External factors, such as peer pressure influencing dietary choices (Ragelienė & Grønhøj, 2020), coupled with socio-cultural influences (e.g., prevalent advertising of unhealthy foods in metropolitan areas (Richmond et al., 2020, Sainsbury et al., 2017)) may have counteracted the positive intentions fostered by the intervention, making it more challenging for these adolescents to adopt healthier eating habits. Future interventions should focus on modifying behavioural intentions and strategies to mitigate these external influences.

Unexpectedly, regional participants in the intervention group had greater growth in the odds of having a poor diet compared to controls. This disparity may be due to the health education provided in regional control groups placing a stronger emphasis on dietary education than *Health4Life*. While *Health4Life*, given its multiple behaviour change framework, provided an equivalent of one to two 15-minute lessons on diet, teacher-reported data revealed that most regional control schools dedicated a minimum of one to two 40-minute lessons solely to diet education, and some delivered up to six lessons. Within the Australian curriculum, students learn about making healthy and safe food choices, including food-serving recommendations from *The Australian Guide to Healthy Eating* and practical advice on choosing healthy options from the school canteen (ACARA Version 9.0)*.* The control schools’ focused approach, coupled with greater frequency and depth on diet education than *Health4Life*, may have equipped students with more knowledge and strategies for making healthier food choices. This suggests *Health4Life* should incorporate a more intensive dietary education component, especially in regional areas. Although it is worth noting that data were not collected on other characteristics in the control and *Health4Life* schools, such as policies (canteen, nutrition, other health promotion policies) and food environments, which may have affected diet outcomes. Nevertheless, as previously mentioned, *Health4Life* was not specifically designed for disadvantaged adolescents, and regional areas may have unique challenges that the intervention did not adequately address. For instance, control schools may have better addressed the social and environmental context, including lower affordability or availability of healthy food, to ensure health-promoting messages were relevant, practical, and accessible within the local context. To overcome this, future studies could adopt matched sampling methods to ensure the control and intervention schools do not drastically differ in the amount of health education they deliver (Chondros et al., 2021, Hemming and Taljaard, 2023). A process evaluation of *Health4Life* in regional schools is also needed to understand the implementation context and identify any other factors that may have contributed to this unintended effect. Ultimately, the conflicting diet-related findings observed in this study highlight the need for further research to replicate and explore potential explanations, informing improvements in future interventions targeting dietary behaviours among disadvantaged adolescents.

### 4.1 Strengths and limitations

This study has several strengths and limitations. Firstly, the use of the composite diet risk score provides an overall perspective on diet, however, may overlook specific dietary nuances. Although the fruit and vegetable variables align with official government guidelines (National Health and Medical Research Council, 2013), the lack of guidelines for SSB and junk food variables required input from a multidisciplinary team, including nutritionists, and following health recommendations to determine the “at-risk” cutoffs for these variables. Additionally, the item assessing diet-related intentions focused solely on intentions to swap SSB for water. Although SSB is one relevant indicator of poor diet (National Health and Medical Research Council, 2013), future studies should adopt more comprehensive measures.

The use of the Family Affluence Scale III as a proxy measure for SES, while practical, simplifies evaluation by relying on a limited number of indicators, such as overseas holidays (Torsheim et al., 2016). This approach may overlook the multidimensional aspects of SES, including income and education. Furthermore, self-reported information introduces potential bias, as respondents may interpret scale items differently. Acknowledging limitations imposed by the COVID-19 pandemic is also essential. In Australia, adolescents in high and low lockdown states experienced increased negative emotions (Meyer et al., 2023), poorer mental health, limited physical activity and increased screen time (Gardner et al., 2022b, Goldfeld et al., 2022). Similar patterns were observed overseas (Lawrance et al., 2022). Although *Health4Life* was implemented in 2019, the follow-up assessments coincided with the pandemic, possibly influencing participants’ response to *Health4Life* and their ability to apply acquired knowledge and skills.

The study sample included students from independent, public, and Catholic secondary schools across three Australian states, contributing to its diversity. However, most students were born in Australia, of middle or upper SES, and residing in major cities (Champion et al., 2021), potentially limiting generalisability. Moreover, including only inner and outer regional students, with no remote schools, impacts understanding whether *Health4Life* had any differential effects by diverse regional groups. Finally, geographical moderation analysis results for alcohol and tobacco outcomes require cautious interpretation due to wide confidence intervals, primarily attributed to low cell counts and low prevalence of use.

Notwithstanding these limitations, a key strength of the *Health4Life* study is its rigorous cluster RCT design and large sample size, making it one of the most extensive school-based studies of its nature. The meticulous development and implementation of *Health4Life* involved close collaboration with end-users (Champion et al., 2023), ensuring the intervention’s relevance and engagement among students and teachers. Moreover, *Health4Life*’s favourable acceptability within a school setting enhances its potential for wider applicability and impact. Future work is underway to refine *Health4Life*’s content to better address the specific needs of low SES and geographically remote adolescents.

## 5 Conclusions

This study offers valuable insights into how SES and geographical factors interacted with the *Health4Life* intervention’s effectiveness in targeting alcohol and tobacco use, dietary intake, knowledge, behavioural intentions, and psychological distress. Generally, outcomes were not influenced by SES and geographical location. However, the exception to this pattern is in diet-related outcomes, with varying effects on poor diet and diet-related behavioural intentions based on participants’ geographical location, underscoring the importance of considering regional differences in intervention design and implementation. Co-designing and tailoring interventions is essential to address disparities in chronic disease risk behaviours among disadvantaged adolescent populations. Future research should consider the impact of SES and geographical factors in intervention design and implementation to optimise outcomes and promote health equity.

## Funding

This work was supported by the Paul Ramsay Foundation and the Australian National Health and Medical Research Council (Fellowships granted to KEC [APP1120641], NCN [APP1166377]. The funders were not involved in the study design, implementation or publication process.

## CRediT authorship contribution statement

**Lyra Egan:** Writing – review & editing, Writing – original draft, Visualization, Software, Methodology, Formal analysis, Conceptualization. **Lauren A. Gardner:** Writing – review & editing, Supervision, Project administration, Investigation, Conceptualization. **Nicola C. Newton:** Writing – review & editing, Supervision, Project administration, Funding acquisition, Conceptualization. **Siobhan O’Dean:** Writing – review & editing, Validation, Software, Methodology, Formal analysis, Data curation. **Katrina E. Champion:** Writing – review & editing, Supervision, Project administration, Funding acquisition, Conceptualization.

## Declaration of competing interest

The authors declare that they have no known competing financial interests or personal relationships that could have appeared to influence the work reported in this paper.

## Data Availability

The study’s statistical analysis code (syntax) and the collected data, which has been de-identified to protect participants’ privacy, will be made accessible to other researchers upon request to the corresponding author. Such requests should be accompanied by a valid study protocol and analysis plan. Data sharing will be facilitated after the approval of a proposal by a committee within the current research team, with a signed data access agreement. The informed consent forms can be found in the published protocol (Teesson et al., 2020).
