# Supplementary material for: Moderating effects of socioeconomic status and geographical location on the Health4Life school-based intervention
Source: Prev Med Rep. 2024 Aug 13;46:102855. doi: 10.1016/j.pmedr.2024.102855 (PMC11378973; doi:10.1016/j.pmedr.2024.102855)
Supplement: Supplementary Data 1 [file mmc1.docx]

Supplementary materials

Table of Contents

[**Appendix Figure 1. Consolidated Standards of Reporting Trials (CONSORT) summary of participant flow through the Health4Life study** 2](#_Toc155776439)

[**Measures: additional information** 3](#_Toc155776441)

[**Statistical Analyses: Description of Model Types** 7](#_Toc155776442)

[**Statistical Analyses: Model Fit** 8](#_Toc155776443)

[**Appendix Table 1.** Model fit statistics for the best fitting unconditional growth models 8](#_Toc155776444)

[**Appendix Table 2. Summary of raw number of participants and prevalence (95% confidence interval) for each categorical outcome by time, intervention status and participant characteristics** 9](#_Toc155776445)

[**Appendix Table 3. Summary of raw data for each continuous outcome by time, intervention status and participant characteristics** 12](#_Toc155776446)

[**Appendix Table 4*.* Geographical location subgroup analyses on moderating the odds of diet-related outcomes** 13](#_Toc155776447)

Appendix Figure 1. Consolidated Standards of Reporting Trials (CONSORT) summary of participant flow through the Health4Life study (Champion et al., 2023)

Invited to participate:

519 schools

**Enrolment**

EnrollmentEnrolment

Excluded (not meeting inclusion criteria*):

434 schools

Excluded (not meeting inclusion criteria*):

434 schools

Recruited: 85 schools

(9280 eligible students)

Recruited: 85 schools

(9280 eligible students)

**Allocation**

**Allocation**

Control group: 43 schools

(3421 of 4704 students with parental consent)

Control group: 43 schools

(3421 of 4704 students with parental consent)

Health4Life group: 42 schools

(3743 of 4576 students with parental consent)

Health4Life group: 42 schools

(3743 of 4576 students with parental consent)

Withdrew (lack of time): 5 schools

Excluded from baseline sample: 1 school

Withdrew (lack of time): 5 schools

Excluded from baseline sample: 1 school

Withdrew (lack of time): 7 schools

Excluded from baseline sample: 1 school

Withdrew (lack of time): 7 schools

Excluded from baseline sample: 1 school

**Baseline**

**Baseline**

Baseline completion

36 schools (3609 students; 54.4%)

Baseline completion

36 schools (3609 students; 54.4%)

Baseline completion

35 schools (3030 students; 45.6%)

Baseline completion

35 schools (3030 students; 45.6%)

**Follow-up assessments**

**Follow-up assessments**

Assessed:

Post-test: 35 schools (2665 students; 90.0%)

12-months: 35 schools (2577 students; 85.0%)

24-months: 35 schools (2367 students; 78.1%)

Assessed:

Post-test: 35 schools (2665 students; 90.0%)

12-months: 35 schools (2577 students; 85.0%)

24-months: 35 schools (2367 students; 78.1%)

Assessed:

Post-test: 36 schools (3303 students; 91.5%)

12-months: 36 schools (2948 students; 81.7%)

24-months: 36 schools (2647 students; 73.3%)

Assessed:

Post-test: 36 schools (3303 students; 91.5%)

12-months: 36 schools (2948 students; 81.7%)

24-months: 36 schools (2647 students; 73.3%)

Analysed:

35 schools (3030 students) included in final analysis)

Analysed:

35 schools (3030 students) included in final analysis)

Analysed:

36 schools (3609 students) included in final analysis)

**Analysis**Analysed:

36 schools (3609 students) included in final analysis)

**Analysis**

**AnalysisAnalysis**

# Analysis

*schools were excluded if they: (1) had fewer than 30 year 7 students; (2) relevant ethics approval was not obtained; and (3) were not located in Greater Sydney (NSW), regional areas of New South Wales, within a 100 km radius from Brisbane (QLD), or within a 600 km radius from Perth (WA).

# Measures: additional information

Sociodemographic factors

SES: Students provided self-reported information on gender, age, SES and geographical location. To assess SES, the Family Affluence Scale III was used as a proxy measure, which has been widely used and validated in previous studies (Currie et al., 2008; Torsheim et al., 2016). This scale enables a comparison of participants’ SES relative to others within the sample. To generate a comprehensive representation of SES in this study, the scale was transformed into ridit scores ranging from 0 to 1. Higher ridit scores indicate higher relative SES. For the purpose of interpretation, the continuous ridit scores were further categorised into three groups based on the distribution of SES in the study population. These categories included a lower SES group (ridit < 0.2), a middle SES group (ridit ≥ 0.2 and ≤ 0.6), and an upper SES group (ridit > 0.6) (Elgar et al., 2017).

Geographical location: The geographical remoteness of the participants’ schools was assessed using the nationally recognised Australian Statistical Geography Standard Remoteness Structure (Australian Bureau of Statistics, 2021). This classification system categorised schools into major city, inner regional, outer regional, remote, or very remote based on their geographical location. Given that many participants were unsure of their home postcode, the school's geographical remoteness classification was used as a substitute for each individual's geographical remoteness. Schools were then coded in this study as being either metropolitan or regional.

Primary outcomes

Diet: The Student Physical Activity and Nutrition Survey (SPANS) was used to measure consumption of SSBs, fruit, vegetable, and discretionary foods (junk food such as fried potato products, confectionary foods, and takeaway meals) (Hardy et al., 2016). To evaluate the overall risk associated with poor dietary patterns, a composite indicator of poor diet was calculated. Individuals who fulfilled the criteria for high sugar-sweetened beverage (SSB) consumption (5-6 cups/week or 1 or more cups/day of SSB) or met two or more of the following conditions were classified as having a poor diet: consuming fewer than two servings of fruit per day, consuming fewer than five servings of vegetables per day, or consuming more than one serving of discretionary food items per day. The cut-offs for fruit and vegetable intake were established according to the Australian Dietary Guidelines (National Health and Medical Research Council, 2013), while guidance from nutritionists and health recommendations were followed for SSB and discretionary food variables, as official guidelines were not available for these particular variables.

Alcohol: Alcohol consumption was evaluated using a single item measure designed to determine participants' consumption of a full standard drink over the previous six months, where participants were asked, “Have you had a full standard alcoholic drink in the past 6 months?” (0 = No, 1 = Yes). To facilitate participants’ responses, they were shown a pictorial chart displaying the standard drink quantities of various types of alcoholic beverages and sizes.

Tobacco: Tobacco use was assessed using a single item measure from the Youth Risk Behaviour Survey, with participants asked, “In the past 6 months, have you tried cigarette smoking, even one or two puffs?” (0 = No, 1=Yes) (Brener et al., 2013).

Secondary outcomes

Knowledge: A 20-item scale was used to assess participants’ knowledge of the content covered in the *Health4Life* study. This scale was specifically designed to evaluate awareness of chronic disease risk factors and covered important areas such as alcohol guidelines, dietary intake, physical activity, screen time, and sleep. Additionally, the scale encompassed knowledge of the prevalence of alcohol and tobacco use among young Australians, as well as the impact of the six lifestyle risk behaviours targeted in the intervention on both physical and mental health. The items were presented as “True”, “False”, “Don’t Know” statements, and scores were totalled to produce an overall knowledge score.

Behavioural Intentions: Behavioural intentions to participate in or modify behaviours relating to alcohol use, tobacco smoking and poor diet were evaluated using a self-report questionnaire. Alcohol intentions were assessed using established measures (Newton et al., 2012), while items for tobacco and poor diet (specifically sugar-sweetened beverages such as energy drinks, soft drinks, sports drinks, or cordial) were adapted from these measures. Participants rated their likelihood of trying alcohol and tobacco in the future, with responses ranging from 0 (very unlikely) to 4 (very likely). For sugar-sweetened beverages, participants indicated their intention to replace energy drinks, soft drinks, sports drinks, or cordial with water on a scale from 0 (not at all true of me) to 3 (very true of me) on all or most days of the week for the next three months. For subsequent analysis in this study aimed at determining participants' intentions regarding alcohol use, tobacco smoking, and dietary habits, responses were converted into binary variables. For alcohol and tobacco questions, scores of 0-2 indicated no intention to engage in alcohol use or tobacco smoking, while scores of 3-4 indicated an intention to do so. Regarding SSBs, responses of 0-1 were recoded as no intention to replace SSB consumption with water, while responses of 2-3 indicated an intention to make the substitution

Psychological Distress: Psychological distress was assessed using the widely used and validated measure for adolescents, the Kessler 6 (K6) scale, which measures the frequency of six symptoms of psychological distress experienced by individuals over the past four weeks (Furukawa et al., 2003; Mewton et al., 2016). These symptoms include feeling nervous, hopeless, or restless, and others. Participants were asked to rate each symptom on a 5-point Likert scale ranging from “none of the time” to “all of the time”. Scores from the K6 are totalled to create a composite score, with higher scores indicating greater psychological distress.

# Statistical Analyses: Description of Model Types

Latent growth curve models (LGMs) in Mplus (v 8.4. (Muthén & Muthén, 2017)) were used to evaluate the moderating effects of SES and geographical location on primary and secondary outcomes over 24-month post baseline assessments. LGMs are part of the broader structural equation modelling framework that allow for estimating changes in measured variables over time by modelling them as latent variables that are expected to vary over time. A set of growth parameters are included in the model, such as the intercept and slope, which indicate the starting level of the variable at the beginning of the study and the rate of change in the variable over time, respectively. To determine the moderation effect of SES and geographical location on the intervention effectiveness, the relationship between each of these variables and the slope latent factor was examined, yielding an estimation of the difference in the change in outcome over time between varying levels of SES and geographical location. When significant moderation effects were reported (p<0.05), we conducted subset analyses to determine the difference in intervention effects by group, by comparing the varying levels of SES (three levels; 1 = low, 2 = mid, 3 = high) and geographical location (two levels; 0 = metropolitan, 1 = regional) on outcomes.

**Logistic latent growth models:** For the binary variables (poor diet, alcohol and tobacco use, and behavioural intentions) the growth models employed a log odds function to estimate the relationship. The intervention effect sizes, including odds ratios and corresponding 95% CIs were estimated by exponentiating new parameter variables in R Studio. The intervention effect was estimated over a one-year period for the linear models (poor diet, behavioural intentions) and over a 24-month period for the free model (alcohol and tobacco use), during the trial.

**Continuous latent growth models:** For continuous variables (knowledge and psychological distress), the models estimated a mean change and standard error in scores (referred to as the slope parameters). The slope parameters varied depending on the type of model employed, with free applied to knowledge and linear applied to psychological distress. These parameters measured the extent of change in the predicted outcome over a one-year period for the linear model and a 24-month period for the free model, during the trial.

# Statistical Analyses: Model Fit

We tested various model specifications, including linear, quadratic, and freely estimated, on unconditional LGMs to determine the optimal time structure and slope estimates for each outcome. Model selection depended on model fit statistics, including Akaike information criterion (AIC), Bayesian information criterion (BIC), and sample-size adjusted BIC. Based on these statistics, models were either linear estimated time scores (representing average 12-month change) or free (representing average 24-month change). Refer to supplementary table S1 for detailed model fit statistics.

## **Appendix Table 1.** Model fit statistics for the best fitting unconditional growth models

| Variable | Model | χ2 | df | p-value | AIC | BIC | SSABIC | RMSEA | CFI | TLI |
| --- | --- | --- | --- | --- | --- | --- | --- | --- | --- | --- |
| Poor diet | Linear | 8.611 | 10 | 0.5694 | 5067.551 | 5093.839 | 5077.956 |  |  |  |
| Alcohol use in the past 6 months | Free | 4.695 | 8 | 0.7897 | 9309.035 | 9356.581 | 9334.336 |  |  |  |
| Tobacco use in the past 6 months | Free | 7.877 | 7 | 0.4456 | 5323.383 | 5370.918 | 5348.674 |  |  |  |
| Diet intentions | Linear | 36.412 | 10 | <0.01 | 25964.77 | 25998.72 | 25982.83 |  |  |  |
| Alcohol intentions | Linear | 20.929 | 10 | 0.0216 | 24477.253 | 24511.206 | 24495.317 |  |  |  |
| Tobacco intentions | Linear | 14.648 | 10 | 0.1454 | 7062.347 | 7096.298 | 7080.409 |  |  |  |
| Knowledge | Free | 241.727 | 5 | <.001 | 115135.666 | 115196.86 | 115168.26 | 0.085 (95% CI 0.076, 0.094) | 0.960 | 0.952 |
| Psychological distress symptoms over the past four weeks K6 scale | Linear | 55.403 | 5 | <0.01 | 131651.039 | 131712.153 | 131683.553 | 0.039 (95% CI 0.030, 0.049) | 0.981 | 0.977 |

# Appendix Table 2. Summary of raw number of participants and prevalence (95% confidence interval) for each categorical outcome by time, intervention status and participant characteristics

| **Outcomes** | **Number and prevalence (95% CI)** | | | | | | | |
| --- | --- | --- | --- | --- | --- | --- | --- | --- |
|  | **Baseline** | | **Post-intervention follow-up** | | **12-month follow-up** | | **24-month follow-up** | |
| **Poor diet** |  |  |  |  |  |  |  |  |
| Low SES |  |  |  |  |  |  |  |  |
| Health4Life | **293/542**  54.1% (49.8-58.2) | | **196/398**  49.2% (44.4-54.1) | | **202/378**  53.4% (48.4-58.4) | | **197/344**  57.3% (52.0-62.4) | |
| Control | **155/316**  49.1% (43.6-54.5) | | **118/270**  43.7% (37.9-49.7) | | **134/267**  50.2% (44.2-56.1) | | **135/256**  52.7% (46.6-58.8) | |
| Mid SES |  |  |  |  |  |  |  |  |
| Health4Life | **632/1156**  54.7% (51.8-57.5) | | **442/940**  47.0% (43.8-50.2) | | **448/860**  52.1% (48.8-55.4) | | **458/802**  57.1% (53.7-60.5) | |
| Control | **441/929**  47.5% (44.3-50.7) | | **382/803**  47.6% (44.1-51.0) | | **398/789**  50.4% (47.0-53.9) | | **376/738**  50.9% (47.3-54.5) | |
| High SES |  |  |  |  |  |  |  |  |
| Health4Life | **735/1504**  48.9% (46.3-51.4) | | **536/1215**  44.1% (41.3-46.9) | | **588/1181**  49.8% (46.9-52.6) | | **556/1012**  54.9% (51.9-58.0) | |
| Control | **561/1211**  46.3% (43.5-49.1) | | 487/1108  44.0% (41.1-46.9) | | **493/1031**  47.8% (44.8-50.9) | | **501/968**  51.8% (48.6-54.9) | |
| Metropolitan |  |  |  |  |  |  |  |  |
| Health4Life | **1595/3039**  52.5% (50.7-54.3) | | **1187/2561**  46.3% (44.4-48.3) | | **1217/2366**  51.4% (49.4-53.4) | | **1197/2133**  56.1% (54.0-58.2) | |
| Control | **1078/2209**  48.8% (46.7-50.9) | | **990/2096**  47.2% (45.1-49.4) | | **1035/2017**  51.3% (49.1-53.5) | | **1034/1939**  53.3% (51.1-55.5) | |
| Regional |  |  |  |  |  |  |  |  |
| Health4Life | **119/245**  48.6% (42.4-54.8) | | **75/155**  48.4% (40.7-56.2) | | **108/190**  56.8% (49.7-63.7) | | **95/157**  60.5% (52.7-67.8) | |
| Control | **128/321**  39.9% (34.7-45.3) | | **100/270**  37.0% (31.5-42.9) | | **102/280**  36.4% (31.0-42.2) | | **96/227**  42.3% (36.0-48.8) | |
| **Alcohol use in the past**  **6 months** |  |  |  |  |  |  |  |  |
| Low SES |  |  |  |  |  |  |  |  |
| Health4Life | **31/558**  5.6% (3.9-7.8) | | **14/421**  3.3% (2.0-5.5) | | **50/425**  11.8% (9.0-15.2) | | **62/370**  16.8% (13.3-20.9) | |
| Control | **10/351**  2.8% (1.6-5.2) | | **16/286**  5.6% (3.5-8.9) | | **22/280**  7.9% (5.2-11.6) | | **33/264**  12.5% (9.0-17.0) | |
| Mid SES |  |  |  |  |  |  |  |  |
| Health4Life | **26/1202**  2.2% (1.5-3.2) | | **18/984**  1.8% (1.2-2.9) | | **68/943**  7.2% (5.7-9.0) | | **148/854**  17.3% (14.9-20.0) | |
| Control | **23/1003**  2.3% (1.5-3.4) | | **33/830**  4.0% (2.8-5.5) | | **58/844**  6.9% (5.4-8.8) | | **115/775**  14.8% (12.5-17.5) | |
| High SES |  |  |  |  |  |  |  |  |
| Health4Life | **46/1559**  3.0% (2.2-3.9) | | **47/1270**  3.7% (2.8-4.9) | | **99/1263**  7.8% (6.5-9.5) | | **178/1068**  16.7% (14.5-19.0) | |
| Control | **31/1336**  2.3% (1.6-3.3) | | **41/1150**  3.6% (2.6-4.8) | | **94/1115**  8.4% (6.9-10.2) | | **158/1007**  15.7% (13.6-18.1) | |
| Metropolitan |  |  |  |  |  |  |  |  |
| Health4Life | **86/3203**  2.7% (2.2-3.3) | | **75/2683**  3.0% (2.2-3.5) | | **194/2590**  7.5% (6.5-8.6) | | **352/2264**  15.5% (14.1-17.1) | |
| Control | **59/2520**  2.3% (1.8-3.0) | | **82/2195**  3.7% (3.0-4.6) | | **167/2171**  7.7% (6.6-8.9) | | **301/2009**  15.0% (13.5-16.6) | |
| Regional |  |  |  |  |  |  |  |  |
| Health4Life | **24/263**  9.1% (6.2-13.2) | | **9/163**  5.5% (2.9-10.2) | | **37/214**  17.3% (12.8-22.9) | | **56/174**  32.2% (25.7-39.4) | |
| Control | **12/359**  3.3% (1.9-5.8) | | **16/282**  5.7% (3.5-9.0) | | **23/309**  7.4% (5.0-10.9) | | **41/255**  16.1% (12.1-21.1) | |
| **Tobacco use in the past**  **6 months** |  |  |  |  |  |  |  |  |
| Low SES |  |  |  |  |  |  |  |  |
| Health4Life | **16/558**  2.9% (1.8-4.6) | | **14/419**  3.3% (2.0-5.5) | | **19/422**  4.5% (2.9-6.9) | | **30/369**  8.1% (5.8-11.4) | |
| Control | **15/349**  4.3% (2.6-7.0) | | **10/286**  3.5% (1.9-6.3) | | **11/280**  3.9% (2.2-6.9) | | **14/265**  5.3% (3.2-8.7) | |
| Mid SES |  |  |  |  |  |  |  |  |
| Health4Life | **15/1200**  1.3% (0.8-2.1) | | **21/980**  2.1% (1.4-3.3) | | **42/934**  4.5% (3.3-6.0) | | **58/852**  6.8% (5.3-8.7) | |
| Control | **19/1002**  1.9% (1.2-2.9) | | **15/833**  1.8% (1.1-2.9) | | **29/842**  3.4% (2.4-4.9) | | **34/774**  4.4% (3.2-6.1) | |
| High SES |  |  |  |  |  |  |  |  |
| Health4Life | **19/1560**  1.2% (0.8-1.9) | | **19/1269**  1.5% (0.9-2.3) | | **43/1259**  3.4% (2.5-4.6) | | **70/1059**  6.6% (5.3-8.3) | |
| Control | **7/1333**  0.5% (0.3-1.1) | | **18/1146**  1.6% (0.9-2.5) | | **48/1112**  4.3% (3.3-5.7) | | **62/1002**  6.2% (4.9-7.9) | |
| Metropolitan |  |  |  |  |  |  |  |  |
| Health4Life | **45/2192**  1.4% (1.1-1.9) | | **46/2674**  1.7% (1.3-2.3) | | **92/2573**  3.6% (2.9-4.4) | | **134/2250**  6.0% (5.1-7.0) | |
| Control | **38/2495**  1.5% (1.1-2.1) | | **41/2191**  1.9% (1.4-2.5) | | **84/2162**  3.9% (3.1-4.8) | | **109/2005**  5.4% (4.5-6.5) | |
| Regional |  |  |  |  |  |  |  |  |
| Health4Life | **9/260**  3.5% (1.8-6.4) | | **6/162**  3.7% (1.7-7.8) | | **16/208**  7.7% (4.8-12.1) | | **30/173**  17.3% (12.4-23.7) | |
| Control | **5/358**  1.4% (0.6-3.2) | | **7/282**  2.5% (1.2-5.0) | | **11/308**  3.6% (2.0-6.3) | | **12/253**  4.7% (2.7-8.1) | |
| **Diet intentions** |  |  |  |  |  |  |  |  |
| Low SES |  |  |  |  |  |  |  |  |
| Health4Life | **348/556**  62.6% (58.5-66.5) | | **283/415**  68.2% (63.6-72.5) | | **262/415**  63.1% (58.4-67.6) | | **256/367**  69.8% (64.9-74.2) | |
| Control | **219/346**  63.3% (58.1-68.2) | | **193/284**  68.0% (62.3-73.1) | | **170/276**  61.6% (55.7-67.1) | | **193/259**  74.5% (68.9-79.4) | |
| Mid SES |  |  |  |  |  |  |  |  |
| Health4Life | **754/1194**  63.1% (60.4-65.8) | | **690/972**  71.0% (68.1-73.8) | | **630/930**  67.7% (64.7-70.6) | | **593/839**  70.7% (67.5-73.7) | |
| Control | **666/1001**  66.5% (63.6-69.4) | | **590/828**  71.3% (68.1-74.2) | | **547/835**  65.5% (62.2-68.7) | | **524/770**  68.1% (64.7-71.2) | |
| High SES |  |  |  |  |  |  |  |  |
| Health4Life | **979/1553**  63.0% (60.1-65.4) | | **887/1256**  70.6% (68.0-73.1) | | **826/1247**  66.2% (63.6-68.8) | | **745/1052**  70.8% (68.0-73.4) | |
| Control | **848/1328**  63.9% (61.2-66.4) | | **788/1145**  68.8% (66.1-71.4) | | **725/1105**  65.6% (62.8-68.4) | | **674/994**  67.8% (64.8-70.6) | |
| Metropolitan |  |  |  |  |  |  |  |  |
| Health4Life | **1986/3169**  62.7% (61.0-64.3) | | **1858/2651**  70.1% (68.3-71.8) | | **1680/2552**  65.8% (64.0-67.6) | | **1579/2228**  70.9% (68.9-72.7) | |
| Control | **1590/2476**  64.2% (62.3-66.1) | | **1500/2182**  68.7% (66.8-70.7) | | **1385/2145**  64.6% (62.5-66.6) | | **1342/1983**  67.7% (65.6-69.7) | |
| Regional |  |  |  |  |  |  |  |  |
| Health4Life | **158/259**  61.0% (54.9-66.7) | | **106/161**  65.8% (58.2-72.7) | | **126/204**  61.8% (54.9-68.2) | | **103/170**  60.6% (53.1-67.6) | |
| Control | **241/357**  67.5% (62.5-72.2) | | **198/279**  71.0% (65.4-76.0) | | **198/303**  65.3% (59.8-70.5) | | **176/251**  70.1% (64.2-75.4) | |
| **Alcohol intentions** |  |  |  |  |  |  |  |  |
| Low SES |  |  |  |  |  |  |  |  |
| Health4Life | **174/554**  31.4% (27.7-35.4) | | **128/415**  30.8% (26.6-35.4) | | **162/414**  39.1% (34.5-43.9) | | **173/369**  46.9% (41.9-52.0) | |
| Control | **110/346**  31.8% (27.1-36.9) | | **104/285**  36.5% (31.1-42.2) | | **128/279**  45.9% (40.1-51.7) | | **136/266**  51.1% (45.1-57.1) | |
| Mid SES |  |  |  |  |  |  |  |  |
| Health4Life | **442/1200**  36.8% (34.2-39.6) | | **356/974**  36.6% (33.6-39.6) | | **464/932**  49.8% (46.6-53.0) | | **464/849**  54.7% (51.3-58.0) | |
| Control | **407/1001**  40.7% (37.7-43.7) | | **386/828**  46.6% (43.2-50.0) | | **447/838**  53.3% (50.0-56.7) | | **452/771**  58.6% (55.1-62.1) | |
| High SES |  |  |  |  |  |  |  |  |
| Health4Life | **614/1555**  39.5% (37.1-41.9) | | **489/1262**  38.7% (36.1-41.5) | | **639/1257**  50.8% (48.1-53.6) | | **573/1051**  54.5% (51.5-57.5) | |
| Control | **548/1334**  41.1% (38.5-43.7) | | **491/1141**  43.0% (40.2-45.9) | | **593/1109**  53.5% (50.5-56.4) | | **608/997**  61.0% (57.9-64.0) | |
| Metropolitan |  |  |  |  |  |  |  |  |
| Health4Life | **1150/3183**  36.1% (34.5-37.8) | | **951/2658**  35.8% (34.0-37.6) | | **1219/2562**  47.6% (45.7-49.5) | | **1183/2240**  52.8% (50.7-54.9) | |
| Control | **965/2492**  38.7% (36.8-40.7) | | **929/2178**  42.7% (40.6-44.7) | | **1103/2154**  51.2% (49.1-53.3) | | **1155/1995**  57.9% (55.7-60.0) | |
| Regional |  |  |  |  |  |  |  |  |
| Health4Life | **125/261**  47.9% (41.9-53.9) | | **71/162**  43.8% (36.4-51.5) | | **115/207**  55.6% (48.7-62.2) | | **99/172**  57.6% (50.1-64.7) | |
| Control | **167/357**  46.8% (41.7-52.0) | | **132/281**  47.0% (41.2-52.8) | | **178/306**  58.2% (52.6-63.6) | | **159/252**  63.1% (57.0-68.8) | |
| **Tobacco intentions** |  |  |  |  |  |  |  |  |
| Low SES |  |  |  |  |  |  |  |  |
| Health4Life | **14/552**  2.5% (1.5-4.2) | | **13/414**  3.1% (1.8-5.3) | | **24/416**  5.8% (3.9-8.4) | | **37/369**  10.0% (7.4-13.5) | |
| Control | **8/346**  2.3% (1.2-4.5) | | **10/283**  3.5% (1.9-6.4) | | **21/279**  7.5% (5.0-11.2) | | **18/263**  6.8% (4.4-10.6) | |
| Mid SES |  |  |  |  |  |  |  |  |
| Health4Life | **27/1992**  2.3% (1.6-3.3) | | **31/975**  3.2% (2.2-4.5) | | **58/931**  6.2% (4.8-8.0) | | **64/845**  7.6% (6.0-9.6) | |
| Control | **27/992**  2.7% (1.9-3.9) | | **28/828**  2.4% (2.3-4.8) | | **41/837**  4.9% (3.6-6.6) | | **54/771**  7.0% (5.4-9.0) | |
| High SES |  |  |  |  |  |  |  |  |
| Health4Life | **33/1548**  2.1% (1.5-3.0) | | **29/1259**  2.3% (1.6-3.3) | | **67/1250**  5.4% (4.2-6.6) | | **91/1051**  8.6% (7.1-10.5) | |
| Control | **24/1331**  1.8% (1.2-2.7) | | **32/1142**  2.8% (2.0-3.9) | | **69/1105**  6.2% (5.0-7.8) | | **80/994**  8.0% (6.5-9.9) | |
| Metropolitan |  |  |  |  |  |  |  |  |
| Health4Life | **65/3168**  2.1% (1.6-2.6) | | **74/2653**  2.8% (2.2-3.5) | | **137/2556**  5.4% (4.6-6.3) | | **176/2236**  7.9% (6.8-9.1) | |
| Control | **51/2481**  2.1% (1.6-2.7) | | **72/2177**  3.3% (2.6-4.1) | | **130/2149**  6.0% (5.1-7.1) | | **155/1990**  7.8% (6.7-9.0) | |
| Regional |  |  |  |  |  |  |  |  |
| Health4Life | **12/258**  4.7% (2.7-8.0) | | **8/162**  4.9% (2.5-9.4) | | **18/207**  8.7% (5.6-13.3) | | **27/171**  15.8% (11.1-22.0) | |
| Control | **11/356**  3.1% (1.7-5.4) | | **9/280**  3.2% (1.7-6.0) | | **20/306**  6.5% (4.3-9.9) | | **15/251**  6.0% (3.7-9.6) | |

# Appendix Table 3. Summary of raw data for each continuous outcome by time, intervention status and participant characteristics

|  |  | **Mean (95% confidence interval)** | |  |
| --- | --- | --- | --- | --- |
| **Outcome** | **Baseline** | **Post-intervention follow-up** | **12-month follow-up** | **24-month follow-up** |
| **Knowledge** |  |  |  |  |
| Low SES |  |  |  |  |
| Health4Life | 11.4 (11.17-11.71) | 13.3 (12.91-13.74) | 12.4 (11.99-12.72) | 12.4 (12.00-12.81) |
| Control | 11.9 (11.62-12.24) | 12.0 (11.57-12.37) | 12.3 (11.93-12.62) | 12.2 (11.82-12.66) |
| Mid SES |  |  |  |  |
| Health4Life | 12.0 (11.83-12.17) | 14.3 (14.08-14.51) | 13.2 (13.03-13.47) | 13.3 (13.05-13.49) |
| Control | 12.4 (12.25-12.62) | 12.7 (12.49-12.93) | 13.0 (12.75-13.15) | 12.7 (12.51-12.97) |
| High SES |  |  |  |  |
| Health4Life | 12.0 (11.90-12.20) | 14.3 (14.15-14.54) | 13.3 (13.07-13.46) | 13.3 (13.05-13.46) |
| Control | 12.4 (12.26-12.57) | 12.7 (12.53-12.90) | 12.5 (12.34-12.73) | 12.6 (12.37-12.80) |
| Metropolitan |  |  |  |  |
| Health4Life | 11.9 (11.78-11.99) | 14.1 (13.99-14.27) | 13.1 (12.97-13.24) | 13.1 (12.93-13.22) |
| Control | 12.1 (12.01-12.25) | 12.5 (12.32-12.60) | 12.5 (12.33-12.60) | 12.5 (12.32-12.63) |
| Regional |  |  |  |  |
| Health4Life | 12.0 (11.67-12.42) | 13.7 (13.15-14.28) | 12.5 (12.02-12.96) | 12.6 (12.07-13.06) |
| Control | 13.1 (12.77-13.33) | 13.2 (12.80-13.52) | 13.4 (13.04-13.67) | 12.9 (12.55-13.26) |
| **Psychological Distress** |  |  |  |  |
| Low SES |  |  |  |  |
| Health4Life | 7.3 (6.82-7.77) | 7.0 (6.37-7.54) | 8.3 (7.66-8.99) | 8.2 (7.49-8.85) |
| Control | 7.6 (6.97-8.19) | 7.1 (6.39-7.79) | 7.9 (7.11-8.64) | 8.0 (7.27-8.82) |
| Mid SES |  |  |  |  |
| Health4Life | 6.8 (6.49-7.11) | 6.2 (5.79-6.53) | 7.3 (6.89-7.68) | 7.7 (7.27-8.12) |
| Control | 6.9 (6.59-7.24) | 6.6 (6.18-6.96) | 7.8 (7.41-8.25) | 8.4 (8.00-8.87) |
| High SES |  |  |  |  |
| Health4Life | 6.5 (6.29-6.81) | 6.2 (5.86-6.50) | 7.4 (7.06-7.75) | 8.1 (7.67-8.43) |
| Control | 6.7 (6.45-7.01) | 6.6 (6.28-6.91) | 7.9 (7.57-8.31) | 8.2 (7.79-8.55) |
| Metropolitan |  |  |  |  |
| Health4Life | 6.8 (6.62-7.00) | 6.3 (6.11-6.56) | 7.5 (7.25-7.73) | 7.9 (7.67-8.20) |
| Control | 7.0 (6.79-7.21) | 6.8 (6.57-7.05) | 8.1 (7.82-8.35) | 8.3 (7.99-8.53) |
| Regional |  |  |  |  |
| Health4Life | 6.8 (6.17-7.45) | 6.6 (5.66-7.62) | 7.8 (6.90-8.65) | 7.8 (6.81-8.78) |
| Control | 6.4 (5.89-6.95) | 6.0 (5.30-6.64) | 6.6 (5.98-7.30) | 7.8 (7.02-8.50) |

# Appendix Table 4*.* Geographical location subgroup analyses on moderating the odds of diet-related outcomes

| **Outcome** | **Location** | **Intercept** | | **Slope** | |
| --- | --- | --- | --- | --- | --- |
|  |  | OR (CI) | p | OR (95% CI) | p |
| Poor diet | Metropolitan | 1.08 (0.83-1.42) | 0.565 | 0.99 (0.86-1.14) | 0.915 |
|  | Regional | 1.15 (0.51-2.61) | 0.733 | 1.61 (1.13-2.29) | 0.008 |
|  | | | | | |
| Diet intentions | Metropolitan | 0.98 (0.81-1.19) | 0.866 | 1.13 (1.01-1.27) | 0.041 |
|  | Regional | 1.77 (0.53-0.97) | 0.030 | 0.97 (0.72-1.32) | 0.857 |

References

Australian Bureau of Statistics. (2021). *Australian Statistical Geography Standard (ASGS) Edition 3*. ABS. https://www.abs.gov.au/statistics/standards/australian-statistical-geography-standard-asgs-edition-3/jul2021-jun2026/remoteness-structure#cite-window1

Brener, N. D., Kann, L., Shanklin, S., Kinchen, S., Eaton, D. K., Hawkins, J., & Flint, K. H. (2013). Methodology of the youth risk behavior surveillance system - 2013. *Morbidity and Mortality Weekly Report: Recommendations and Reports*, *62*(1), 1-20.

Champion, K. E., Newton, N. C., Gardner, L. A., Chapman, C., Thornton, L., Slade, T., Sunderland, M., Hides, L., McBride, N., O'Dean, S., Kay-Lambkin, F., Allsop, S., Lubans, D. R., Parmenter, B., Mills, K., Spring, B., Osman, B., Ellem, R., Smout, S., . . . Health4Life Team. (2023). Health4Life eHealth intervention to modify multiple lifestyle risk behaviours among adolescent students in Australia: a cluster-randomised controlled trial. *The Lancet Digital Health*, *5*(5), e276–e287. https://doi.org/10.1016/S2589-7500(23)00028-6

Currie, C., Molcho, M., Boyce, W., Holstein, B., Torsheim, T., & Richter, M. (2008). Researching health inequalities in adolescents: the development of the Health Behaviour in School-Aged Children (HBSC) family affluence scale. *Social Science & Medicine (1982)*, *66*(6), 1429-1436. https://doi.org/10.1016/j.socscimed.2007.11.024

Elgar, F. J., Xie, A., Pförtner, T.-K., White, J., & Pickett, K. E. (2017). *Assessing the View from Bottom: How to Measure Socioeconomic Position and Relative Deprivation in Adolescents*. SAGE Publications Ltd. https://doi.org/10.4135/9781526406347

Furukawa, T. A., Kessler, R. C., Slade, T., & Andrews, G. (2003). The performance of the K6 and K10 screening scales for psychological distress in the Australian National Survey of Mental Health and Well-Being. *Psychological Medicine*, *33*(2), 357-362. https://doi.org/10.1017/S0033291702006700

Hardy, L. L., Mihrshahi, S., Drayton, B. A., & Bauman, A. (2016). *NSW Schools Physical Activity and Nutrition Survey (SPANS) 2015: Full Report*. https://www.health.nsw.gov.au/heal/Publications/spans-2015-full-report.PDF

Mewton, L., Kessler, R. C., Slade, T., Hobbs, M. J., Brownhill, L., Birrell, L., Tonks, Z., Teesson, M., Newton, N., Chapman, C., Allsop, S., Hides, L., McBride, N., & Andrews, G. (2016). The psychometric properties of the kessler psychological distress scale (K6) in a general population sample of adolescents. *Psychological Assessment*, *28*(10), 1232-1242. https://doi.org/10.1037/pas0000239

Muthén, L. K., & Muthén, B. O. (2017). Mplus User's Guide (1998–2017). In (Eighth ed.). Los Angeles, CA, USA: Muthén & Muthén.

National Health and Medical Research Council. (2013). *Australian Dietary Guidelines*.

Newton, N. C., Teesson, M., Barrett, E. L., Slade, T., & Conrod, P. J. (2012). The CAP study, evaluation of integrated universal and selective prevention strategies for youth alcohol misuse: study protocol of a cluster randomized controlled trial. *BMC Psychiatry*, *12*, 118. https://doi.org/10.1186/1471-244X-12-118

Torsheim, T., Cavallo, F., Levin, K. A., Schnohr, C., Mazur, J., Niclasen, B., Currie, C., & FAS Development Study Group. (2016). Psychometric Validation of the Revised Family Affluence Scale: a Latent Variable Approach. *Child Indicators Research*, *9*, 771-784. https://doi.org/10.1007/s12187-015-9339-x
